# Crystal structure of nitrogen regulatory protein IIA^Ntr ^from *Neisseria meningitidis*

**DOI:** 10.1186/1472-6807-5-13

**Published:** 2005-08-10

**Authors:** Jingshan Ren, Sarah Sainsbury, Nick S Berrow, David Alderton, Joanne E Nettleship, David K Stammers, Nigel J Saunders, Raymond J Owens

**Affiliations:** 1 The Oxford Protein Production Facility, Henry Wellcome Building for Genomic Medicine, University of Oxford, Roosevelt Drive, Oxford, OX3 7BN, UK; 2 Division of Structural Biology, Henry Wellcome Building for Genomic Medicine, University of Oxford, Roosevelt Drive, Oxford, OX3 7BN, UK; 3 The Bacterial Pathogenesis and Functional Genomics Group, The Sir William Dunn School of Pathology, University of Oxford, South Parks Road, Oxford, OX1 3RE, UK

## Abstract

**Background:**

The NMB0736 gene of *Neisseria meningitidis *serogroup B strain MC58 encodes the putative nitrogen regulatory protein, IIA^Ntr ^(abbreviated to NM-IIA^Ntr^). The homologous protein present in *Escherichia coli *is implicated in the control of nitrogen assimilation. As part of a structural proteomics approach to the study of pathogenic *Neisseria *spp., we have selected this protein for structure determination by X-ray crystallography.

**Results:**

The NM-IIA^Ntr ^was over-expressed in *E. coli *and was shown to be partially mono-phosphorylated, as assessed by mass spectrometry of the purified protein.

Crystals of un-phosphorylated protein were obtained and diffraction data collected to 2.5 Å resolution. The structure of NM-IIA^Ntr ^was solved by molecular replacement using the coordinates of the *E. coli *nitrogen regulatory protein IIA^ntr ^[PDB: 1A6J] as the starting model. The overall fold of the *Neisseria *enzyme shows a high degree of similarity to the IIA^Ntr ^from *E. coli*, and the position of the phosphoryl acceptor histidine residue (H67) is conserved. The orientation of an adjacent arginine residue (R69) suggests that it may also be involved in coordinating the phosphate group. Comparison of the structure with that of *E. coli *IIA^mtl ^complexed with HPr [PDB: 1J6T] indicates that NM-IIA^Ntr ^binds in a similar way to the HPr-like enzyme in *Neisseria*.

**Conclusion:**

The structure of NM-IIA^Ntr ^confirms its assignment as a homologue of the IIA^Ntr ^proteins found in a range of other Gram-negative bacteria. We conclude that the NM- IIA^Ntr ^protein functions as part of a phosphorylation cascade which, in contrast to *E. coli*, shares the upstream phosphotransfer protein with the sugar uptake phosphoenolpyruvate:sugar phosphotransferase system (PTS), but in common with *E. coli *has a distinct downstream effector mechanism.

## Background

*Neisseria *spp. are Gram-negative Beta-Protobacteria which include many species found only in humans. Two *Neisseria *spp. are pathogenic to human: *N. meningitidis *and *N. gonorrhoeae*, responsible for bacterial meningitis and septicaemia, and gonorrhoea, respectively. In the last few years, the genomes of *N. meningitidis *serotypes A (strain Z2491) [1] and B (strain MC58) [2] and *N. gonorrhoeae *(strain FA1090) (currently unpublished) have been sequenced and annotated. As part of a structural proteomics approach to the study of pathogenic *Neisseria*, we have solved the structure of the putative nitrogen regulatory protein IIA^Ntr ^of *N. meningitidis *(abbreviated to NM-IIA^Ntr^) (Gene NMB0736), the sequence of which is highly conserved amongst the *Neisseria *spp.

In *Escherichia coli*, the IIA^Ntr ^gene (*ptsN*) is located within the sigma-54 factor coding operon, *rpoN*, with which it is co-transcribed [3]. Insertional mutagenesis of *ptsN *has been shown to suppress the conditional lethality of temperature sensitive *era *(*era*^ts^) mutants [4]. The *era *gene of *E. coli *encodes a GTPase which appears to be essential for cell growth [5]. IIA^Ntr ^is a member of the mannitol-fructose family of IIA protein/domains which forms part of the phosphoenolpyruvate: sugar phosphotransferase system (PTS) [4]. PTS controls sugar uptake by bacteria through a series of phosphoryl transfer reactions in which the sugar-specific IIA enzyme is phosphorylated on a highly conserved histidine residue by the histidine-containing phosphocarrier protein, HPr [6]. In *E. coli*, the *rpoN *operon also includes a gene encoding a protein related to HPr, designated NPr; both HPr and NPr have been shown to phosphorylate *E. coli *IIA^Ntr ^[4]. The sequence similarity between HPr and the molybdenum-iron protein of the nitrogenase complex of *Rhizobium trifolii *[6] and the frequent association of PTS proteins with *rpoN *have been taken to suggest that IIA^Ntr ^may provide a regulatory link between carbon and nitrogen assimilation in bacteria [7].

The role of the NM-IIA^Ntr ^in Neisseria has not been characterized, and the potential functions of these proteins in cellular behaviour are diverse [8]. The gene encoding this protein is not found in proximity to the equivalent of the *E. coli rpoN *gene (NMB0217, which may not be functional) nor is it close to the HPr-like protein (NMB2045). The structures of the *E. coli *IIA^Ntr ^[9] and related IIA^mannitol ^(IIA^mtl^) domain of PTS [10] have been solved by X-ray crystallography. More recently, a solution structure of a phosphoryl transfer complex between IIA^mtl ^domain and HPr has been described [11]. In this report, we compare the structure of the neisserial IIA^Ntr ^to the homologous *E. coli *enzymes and complex and discuss the functional implications of our findings.

## Results and discussion

### Expression and purification

The NM-IIA^Ntr ^was over-expressed in *E. coli *with an N-terminal His-tag which was removed prior to crystallization. Mass spectrometric analysis of the purified protein showed that it comprised a 60:40 mixture of mono-phosphorylated and un-phosphorylated protein. The conserved H67 in the protein was thus capable of phosphorylation by endogenous HPr/NPr from the expression system. Analyses of re-dissolved crystals of the protein by mass spectrometry showed that only the un-phosphorylated form of the protein was crystallized presumably due to labile nature of the phospho-histidine bond in the acidic crystallisation conditions, 0.1 M sodium citrate, pH 4 (data not shown).

### Overall structure

**Figure 1:**
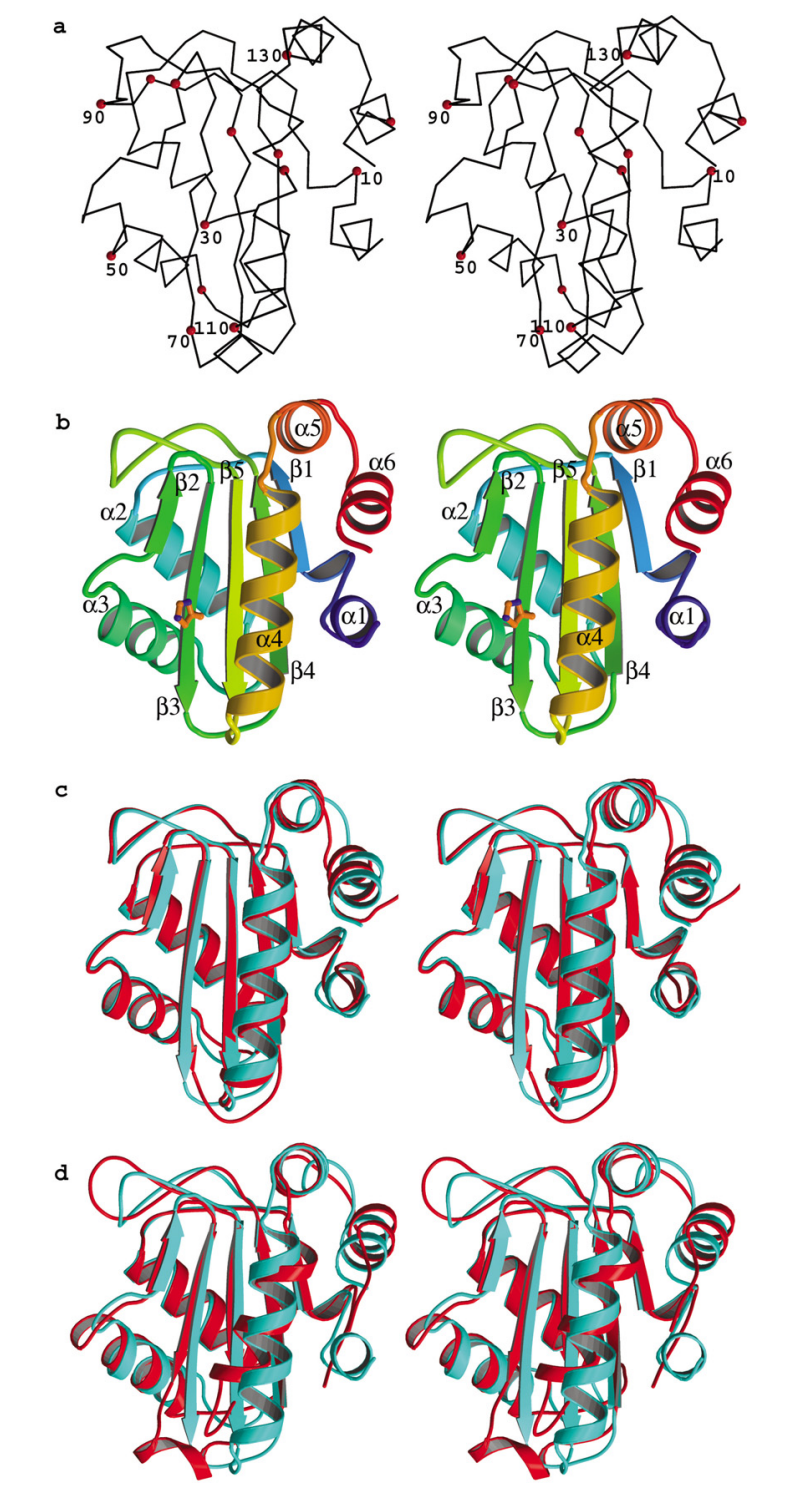
**Structure of NM-IIA^ntr^**. (a) Stereo figure of a C_α_-trace of NM-IIA^Ntr ^with every twentieth residue numbered (b) Stereo figure of a ribbon diagram of NM-IIA^Ntr ^with secondary structure elements labelled (α-helices 1–4; β-strands 1–5). The active site residue H67 is displayed as ball-and-stick. (c) Stereo figure of an overlay of NM-IIA^Ntr ^(green) and *E. coli*IIA^Ntr ^(red) (d) Stereo figure of an overlay of NM-IIA^Ntr^(green) and *E. coli *IIA^mtl^(red).

The model of NM-IIA^Ntr ^contains 146 (out of 149) protein residues and 72 water molecules. Residues 1 and 148–149 are not modelled in the structure due to lack of clearly defined electron density. The structure of NM-IIA^Ntr ^consists of a 5-stranded mixed β-sheet which is sandwiched by six α-helices, two on one side and four on the other (Fig. 1a &1b). The overall fold, as expected from the sequence homology, is similar to that of *E. coli *IIA^Ntr ^(34 % sequence identity) and IIA^mtl ^(23% sequence identity). 122 Cαs out of 150 of *E. coli *IIA^Ntr ^[PDB:1A6J] can be overlapped onto NM-IIA^Ntr ^with a root mean square deviation (rmsd) of 0.78 Å. The major differences between the neisserial and *E. coli *structures appear at surface loops linking the secondary structure elements, for examples α2–α3, β3–β4 and β5-α4 loops (residues 34–40, 70–75 and 107–112 respectively in NM-IIA^Ntr^), and the termini (Fig. 1c). The α1 is shorter and there is a one residue insertion at the α2–α3 loop in NM-IIA^Ntr^. Whilst comparison of *E. coli *IIA^mtl ^[PDB: 1A3A] with NM-IIA^Ntr ^gave 120 equivalent Cαs with an rmsd of 1.48 Å. *E. coli *IIA^mtl ^does not have the short α-helix at the N-terminus, but has a extra helix between β3 and β4. Residue insertions and deletions at α2–α3, β3–β4 and β5-α4 loops have resulted in large structural differences at these places between the two proteins (Fig. 1d). Similar differences between *E. coli *IIA^Nt ^and IIA^mtl ^have been noted [12].

### Active site and interface with H-Pr

**Figure 2:**
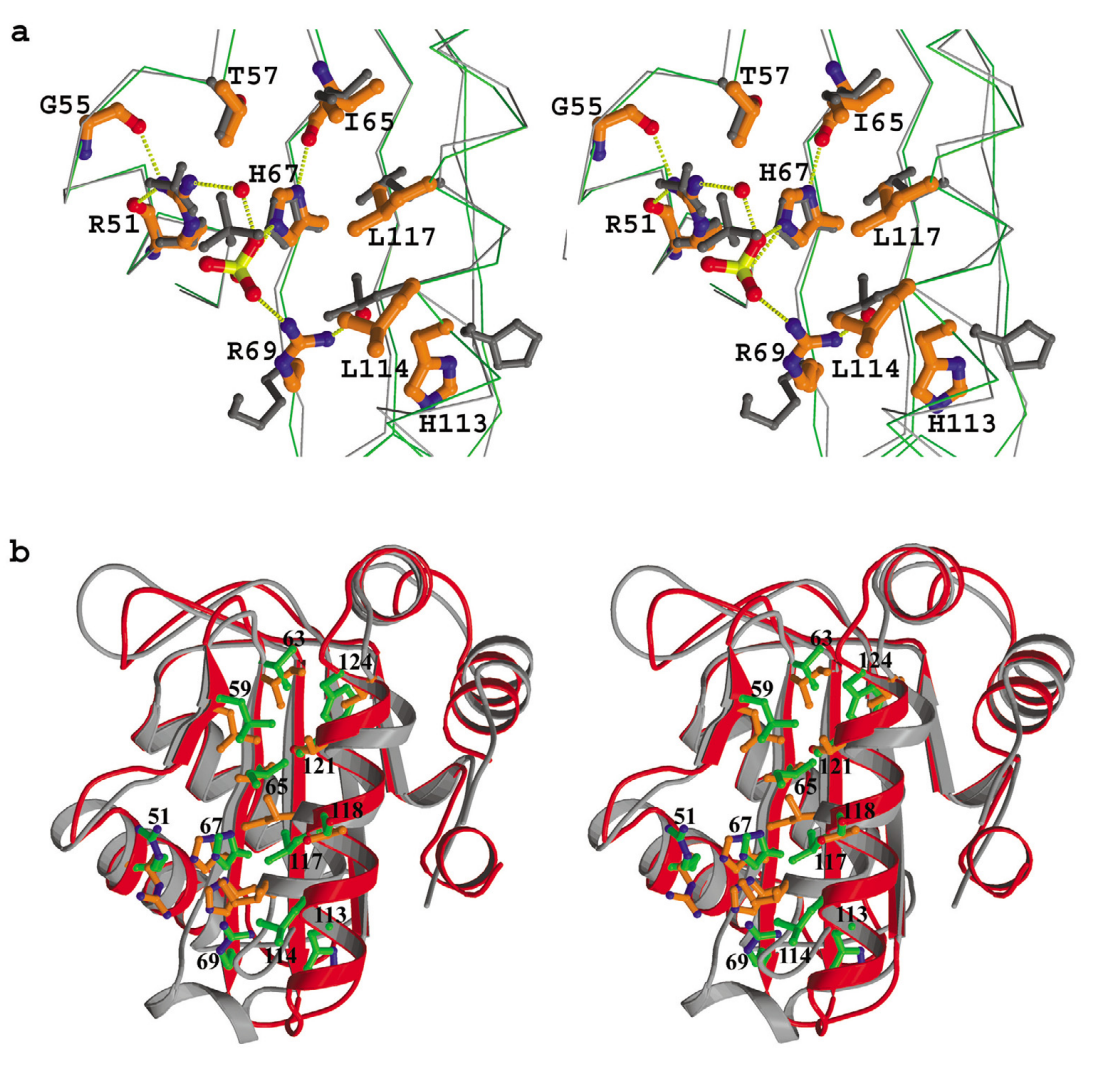
**Active site of NM-IIA^Ntr^**. (a) Stereo figure of an overlay of the active sites of NM-IIA^Ntr ^(green line for main chain and orange for side-chains displayed as ball-and stick) and *E. coli *IIA^mtl ^(grey line for main chain and individual side-chains) showing the positions of residues around the active site H67 and the sulphate ions observed in the structures of both NM-IIA^Ntr ^and *E. coli *IIA^Ntr ^[9]. Residues are numbered according to the sequence of NM-IIA^Ntr^. Hydrogen bonds are shown by broken yellow lines and nitrogen, oxygen and sulphur atoms are displayed as blue, red and yellow respectively. (b) Stereo figure of an overlay of NM-IIA^Ntr ^(red for the main chain ribbon and green for interface residues displayed as ball-and-stick) and *E. coli *IIA^mtl ^(grey for the main chain and orange for interface residues) showing the relative positions of the residues which form the interface between *E. coli *IIA^mtl ^HPr and the corresponding residues in NM-IIA^Ntr^. Residues are numbered according to the sequence of NM-IIA^Ntr^

The active site of NM-IIA^Ntr ^is located in a concave area of protein surface consisting of α3–α4 and β2–β3. The active site residue, H67, the equivalent of H73 in *E. coli *IIA^Ntr ^or H65 in *E. coli *IIA^mtl^, is the fifth residue of β3 and is surrounded by hydrophobic residues L69, I65, L114, L117 and A121 on one side, and by hydrophilic residues R51 and R69 on the other side (Fig. 2a). H67 and R51 are conserved amongst all IIA^Ntr ^and IIA^mtl ^proteins. H67 is stabilized by a hydrogen bond to the carbonyl oxygen of L65 from its ND1 atom, while the NH1 atom of the R51 side-chain hydrogen bonds to the carbonyl groups of both G55 and itself; a similar pattern is also observed in *E. coli *IIA^Ntr^. The side-chain of R69 is folded toward the active site H67 unlike the corresponding K75 of *E. coli *IIA^Ntr^, which is folded away from the active site H73. In the *E. coli *IIA^Ntr ^crystal structure there is a sulphate ion forming a salt bridge/hydrogen bonding interactions to the side-chains of both R57 and H73 in one molecule of the crystal asymmetric unit. Interestingly, in our structure there is also a strong peak of electron density located on the 2-fold crystallographic axis, which could be modelled as a sulphate ion interacting with the side-chains of R51, H67 and R69 via salt bridge/hydrogen bonding, as well as to the equivalent residues of the symmetry related molecule (Fig. 2a). The distance from the ND2 atom of H67 to the two-fold symmetry axis is 3.2 Å, providing insufficient space to accommodate a phosphoryl group, consistent with the finding that only un-phosphorylated protein is found in the crystals by mass spectrometry.

In the active site of the *E. coli *IIA^mtl ^there is a second histidine residue (H111) which is conserved amongst all IIA^mtl ^sequences [13] and is proposed by analogy to the glucose PTS to be essential for phosphoryl transfer to the next component in the cascade, IIB^mtl^[7]. H111 shows two side-chain configurations in both crystal and NMR structures [10,14] either being parallel to and pointing away from, the active site H65 (Fig. 1a). These differing H111 conformations might be related to the two active site geometries required for the phosphoryl group accepting and donating functions of the protein [10]. The equivalent histidine, H113, of NM-IIA^Ntr ^and H120 of *E. coli *IIA^Ntr ^has similar conformations and are located at the back of helix α4 pointing away from the active site. It is unlikely that this histidine can be repositioned at the active site by unwinding the helix and therefore it appears improbable that it play a role in phosphoryl transfer from IIA^Ntr ^to other proteins. The question arises as to whether another residue in the active site of NM-IIA^Ntr ^could play a role in phosphotransfer. Intriguingly, R69 in NM-IIA^Ntr ^is positioned close to the active site and at a similar position to H111 of IIA^mtl ^(Figure 2a). The structure therefore suggests mutagenesis experiments which could be carried out to investigate further the active site of the protein. More generally, it remains an open question as to how NM-IIA^Ntr ^is de-phosphorylated and whether this results in the phosphorylation of another protein(s). Certainly in *E. coli*, IIA^Ntr ^cannot substitute for IIA^mtl ^in the PTS mediated phosphorylation of mannitol [4] and therefore cannot be de-phosphorylated as a consequence of transfer to IIB^mtl^. Since no IIB^Ntr ^component has been identified, it remains unclear what effectors IIA^Ntr ^proteins bind to and what role phosphorylation plays in this interaction.

By contrast, more is known about the protein(s) that interact with IIA^Ntr ^to phosphorylate the protein. Again, results from *E. coli *can be used to inform the likely situation in *Neisseria*. Thus phosphorylation of *E. coli *IIA^Ntr ^involves association with the phospho-transfer proteins HPr, as well as the related NPr, and transfer of a phosphoryl group between histidine residues. HPr and NPr are in turn phosphorylated by the upstream histidine kinases Enzyme I (EI) and EI^Ntr ^respectively, leading to the proposal that there are two parallel phosphoryl transfer chains in *E. coli*, with NPr the preferential donor to IIA^Ntr ^[15]. Interestingly in *Neisseria meningitidis*, there is only one HPr-like protein, encoded by the gene NMB2045. The *N. meningitidis *HPr-like protein (abbreviated to NM-HPr) shares 32% and 38% sequence identity with *E. coli *HPr and NPr respectively. Insight into how IIA proteins bind to the upstream effectors has been obtained by nmr studies. A solution structure has been reported for the complex formed between *E. coli *HPr and IIA^mtl ^[PDB:1J6T] showing that binding is achieved through a central core of hydrophobic contacts strengthened by a few hydrophilic interactions [11]. It is expected that the interactions between the two *Neisseria *proteins should resemble the *E. coli *HPr and IIA^mtl ^complex. Superimposing NM-IIA^Ntr ^onto *E. coli *IIA^mtl ^and aligning the HPr sequence with NM-HPr have indeed revealed common features of protein-protein interactions among the two systems. The key residues of the hydrophobic core of *E. coli *IIA^mtl ^involved in contacts with HPr are L57, I61, I112, I115, T119 and L122, which correspond to residues L59, V63, L114, L117, A121 and F124 in NM-IIA^Ntr^. The aromatic residue F48 of HPr makes most extensive interactions with the hydrophobic core, especially to I61 and L122 of *E. coli *IIA^mtl^, whereas in *Neisseria *the corresponding residues are M48 in NM-HPr, V63 and F124 in IIA^Ntr^, an example of change in shape complementarity. However, since helix α4 of NM-IIA^Ntr ^is 3 residues longer and about 25° different in orientation compared to the same helix of *E. coli *IIA^mtl^, one would anticipate that the NM-HPr could bind in a different orientation. More recently, nmr has been used to characterise the interaction between *E. coli *IIA^Ntr ^and N-Pr [16]. Chemical shift mapping identified the surface on IIA^Ntr ^for NPr binding, which generally corresponds to the HPr -binding region of IIA^mtl ^but specifically implicates G61, D115, S125, T156 and nearby residues in the interaction. The corresponding residues in NM-IIA^Ntr ^are G55, N108, S118 and E149, with the region around G55 being the most highly conserved.

## Conclusion

The structure of NM-IIA^Ntr ^confirms its assignment as a homologue of the IIA^Ntr ^proteins found in a range of other Gram-negative bacteria. In fact the overall fold of the *Neisseria *enzyme shows a high degree of similarity to both the IIA^Ntr ^and IIA^mtl ^proteins from *E. coli*. Further, the orientations of the two histidine residues in the active site region is conserved between the *Neisseria *and *E. coli *IIA^Ntr ^proteins and is distinct from the IIA^mtl^.

The availability of a second IIA^Ntr ^structure enables certain generalizations to be made. The effector mechanism of this sub-group of regulatory proteins is distinct from the IIA components of the PTS controlling sugar uptake, which involve a transfer of phosphate via histidine residues to a IIB acceptor protein. The nature of the downstream effectors of IIA^Ntr ^proteins and the role of IIA^Ntr^phosphorylation in the process remain to be established. In contrast, the mechanism of phosphorylation of all IIA components appears to be broadly similar and involves inter-molecular transfer between histidine residues in a complex formed between HPr and IIA proteins. In *Neisseria *a single HPr-like protein appears to be responsible for phosphorylation of both IIA^Ntr ^and the IIA components of the sugar PTS whereas in *E. coli*, there is a parallel pathway involving the HPr-related protein NPr. In *E. coli*, NPr is expressed, with IIA ^Ntr ^and σ^54^, from the *rpoN *operon implying common regulation of gene expression. This is not the case in *Neisseria *where these genes are found on different transcriptional units. Therefore, although the structure of *Neisseria *IIA^Ntr ^indicates that it is part of a similar phosphotransfer cascade to *E. coli*, details of the regulation of the gene are likely to be distinct.

## Methods

### Protein production

The NM-IIA^Ntr ^expression construct was generated by means of ligation-independent cloning using Gateway™ technology (InVitrogen). The NMB0736 gene [Genbank: AE002098 for complete genome sequence] was amplified from genomic DNA (*Neisseria meningitidis *strain MC58) with KOD HiFi™ polymerase (Novagen) using the forward primer:- 5'ggggacaagtttgtacaaaaaagcaggcttcctggaagttctgttccagggcccgATGAG CCTTATCGGCGAAATTTTG 3' and the following reverse primer:- 5'ggggaccactttgtacaagaaagctgggtctcaTTATTCTTCAGTCAGGATGGCACG 3'

The PCR product was purified using QIAquick 96 plates (Qiagen) and inserted into the vector pDONR221 by recombination between attB (PCR product) and attP (vector) sequences (BP reaction). The insert from this vector was then transferred in to the expression vector pDEST17 by recombination between attL (pDONOR vector) and attR (pDEST vector) sequences (LR reaction). The expression construct contained the following N-terminal His tag and 3C protease cleavage site (underlined) MAHHHHHHAGFLEVLFQGP. BP and LR reactions were carried out according to the manufacturer's instructions. Recombinant LR clones were identified by PCR using a gene specific forward primer and a T7 reverse primer and verified by DNA sequencing. Protein was produced in the *E. coli *strain, B834(DE3). The cells were grown at 37°C in a 1L of GS96 media (QBiogene) to an A_600 _of 0.6, induced with isopropyl β-D-thiogalactopyranoside (IPTG) to 0.5 mM and then incubated for a further 20 h. at 20°C reaching an A_600 _of approximately 5. The cells were harvested by centrifugation at 6000 g for 15 min. and lysed using a Basic-Z Cell Disruptor (Constant Systems Ltd) at 30 Kpsi in 30 ml of 50 mM Tris pH 7.5, containing 500 mM NaCl, 0.2% Tween-20. The NM-IIA^Ntr ^was purified by Nickel affinity chromatography followed by size exclusion chromatography using the standard His Affinity-Gel filtration program on the Akta 3D™ (GE Healthcare). After centrifugation at 30000 g for 30 min., the lysate was loaded onto a 1 ml pre-charged HiTrap ™ Chelating Sepharose™ FF column (GE Healthcare). The column was washed with 50 mM Tris pH7.5, 500 mM NaCl, 20 mM imidazole. The protein was then eluted in 50 mM Tris pH 7.5, 500 mM NaCl, 500 mM imidazole and injected on to a 16/60 HiLoad™ Superdex 200 column (GE Healthcare) equilibrated in 20 mM Tris pH 7.5, 200 mM NaCl. Protein-containing fractions were analysed on SDS-Page gels (Criterion -Biorad). The N-terminal tag was removed by over-night incubation at 4°C with His-tagged 3C protease (prepared from a pET24a/His3C expression vector kindly provided by A. Geerlof, EMBL, Heidelberg). The 3C protease and any uncleaved protein were removed by Nickel affinity chromatography and the protein concentrated to 9.7 mg/ml using a 5 K MWCO Vivaspin 15 concentrator (Vivascience) in 20 mM Tris pH7.5, 200 mM NaCl, 1 mM TCEP. Mass spectrometry of protein and crystals was carried as described [17].

### Crystallization and data collection

The protein was crystallized using the nanodrop crystallization procedure with standard OPPF protocols [18]. Crystals were grown by the sitting drop vapour diffusion method at room temperature from 3.2 M ammonium sulphate, 0.1 M citrate pH 4.0 over a period of 14 days. Diffraction data were collected at station PX14.2 of SRS (Daresbury, UK). Data images were recorded using an ADSC Quantum 4 CCD detector. A crystal mounted in a fibre loop was placed 250 mm from the detector and exposed to the X-ray beam with a wavelength of 0.945 Å. A total of 69 oscillation images of 2.0 degree per exposure were collected from a single crystal frozen under a stream of nitrogen at 100K. The diffraction data were indexed and integrated with DENZO [19] and merged with SCALEPACK. The crystal belongs to the trigonal system, with space group of either *P*3_2_21 or *P*3_1_21 and unit cell dimensions of a = b = 61.02 Å and c = 63.31 Å. There is one molecule in an asymmetric unit, the crystal has a solvent content of 41.5% in the crystal. The data set is 100% complete to 2.5 Å resolution (Table 1).

**Table 1:** X-ray data and refinement statistics

X-ray data	
Space group	P3_1_21
Unit cell dimensions (*a,b,c *in Å)	61.02, 61.02, 63.31
Resolution range (Å)	30.0 - 2.50 (2.59-2.50)^‡^
Unique reflections	4988 (486)
Redundancy	7.6 (5.7)
Completeness (%)	100 (100)
Average *I/σ(I)*	10.2 (2.9)
R_merge_*	0.183 (0.552)
Refinement statistics:	
No. atoms (protein/water)	1105/73
R-factor^†^(R_work_/R_free_)	0.201/0.276
Rms bond length deviation (Å)	0.006
Rms bond angle deviation (°)	1.21
Ramachandran plot statistics	
Residues in most favoured region (%)	87.1
Residues in additional allowed region (%)	12.1
Residues in generously allowed region (%)	0
Residues in disallowed region (%)	0.8 (Arg 35)^#^

^‡ ^data in brackets are for the high resolution shell.

* R_merge _= Σ |*I*-<*I> *|/Σ <*I*> † R-factor = Σ|*F*_*O *_- *F*_*C*_|/Σ *F*_*O*_;

^# ^Arg 35 has well defined main-chain electron density.

### Structure solution and refinement

The structure was solved using molecular replacement with CNS [20] and the coordinates of *E. coli *nitrogen regulatory protein IIA^Ntr ^[PDB:1A6J] as the starting model. The real space cross rotation search was carried out using data from 15–4 Å and Patterson vectors of 5–24 Å. The cross rotation peaks were then subjected to

PC-refinement with e2e2 target followed by translation search with fastf2f2 target. At this stage the space group of the crystal was confirmed to be *P*3_1_21. The highest peak of the translation search (θ_1 _= 150.1, θ_2 _= 68.4, θ_3 _= 82.6, x = 16.5, y = 24.1, z = -35.0), which is 4.2 σ above the mean and 3.2 σ above the 2^nd ^highest one (noise peak), is corresponding to 11^th ^of the 33 cross rotation peaks. Rigid-body refinement of the rotated and translated model at 30-4.0 Å gave an R-factor of 0.462. Rounds of simulated annealing, conjugate gradient minimization and B-factor refinement followed by model rebuilding and solvent molecule addition with O have resulted in the current structure which has a R_work_/R_free _of 0.201/0.276 for all data from 30-2.5 Å resolution. The rms deviation of the model from the ideal is 0.006 Å for bond lengths and 1.21° for bond angles (Table 1).

The atomic coordinates of NM-IIA^Ntr ^and structure factors have been deposited in the Protein Data Bank under the accession code 2A0J.

## Authors' contributions

JR collected and processed the diffraction data, modelled, refined and analyzed the structure, SS purified and crystallized the protein, JN carried out mass spectrometry of the protein and crystals, NB and DA cloned and expressed the protein, NJS initiated the study DKS and RJO coordinated the study. RJO and JR prepared the manuscript with additional input from DKS and NJS.
